# Methylomes of Two Extremely Halophilic *Archaea* Species, Haloarcula marismortui and Haloferax mediterranei

**DOI:** 10.1128/MRA.00577-19

**Published:** 2019-07-03

**Authors:** Shiladitya DasSarma, Alexey Fomenkov, Satyajit L. DasSarma, Tamas Vincze, Priya DasSarma, Richard J. Roberts

**Affiliations:** a Institute of Marine and Environmental Technology, Department of Microbiology and Immunology, University of Maryland—Baltimore, Baltimore, Maryland, USA; b New England Biolabs, Ipswich, Massachusetts, USA; Portland State University

## Abstract

The genomes of two extremely halophilic *Archaea* species, Haloarcula marismortui and Haloferax mediterranei, were sequenced using single-molecule real-time sequencing. The ∼4-Mbp genomes are GC rich with multiple large plasmids and two 4-methyl-cytosine patterns. Methyl transferases were incorporated into the Restriction Enzymes Database (REBASE), and gene annotation was incorporated into the Haloarchaeal Genomes Database (HaloWeb).

## ANNOUNCEMENT

Haloarcula marismortui and Haloferax mediterranei are extremely halophilic microorganisms in the third domain of life forms, the *Archaea*, isolated from hypersaline environments, the Dead Sea and a Spanish saltern, respectively (1, 2). They are of significant interest to the biotechnology industry and the astrobiology community due to their survival in multiple extreme conditions, including saturated salinity, desiccation, high levels of solar radiation, and large numbers of toxic ions (3). We targeted these two halophilic *Archaea* (*Haloarchaea*) species for characterization of methylation patterns and methyl transferases utilizing whole-genome single-molecule real-time (SMRT) sequencing.

Haloarcula marismortui ATCC 43049 and Haloferax mediterranei ATCC 33500 were obtained from the American Type Culture Collection (ATCC) and grown according to the instructions of the ATCC, and nucleic acids were extracted using a published method (4). Sequencing was performed using the PacBio RS II platform with a SMRTbell sequencing library prepared from 3 g genomic DNA from each microbe and randomly sheared to 20 kb using the G-tube protocol (Covaris, Woburn, MA, USA). The libraries were sequenced using two SMRT cells each with P6-C4 chemistry and 360-min collection times. Sequencing reads were filtered (quality, ≥0.80; length, ≥100 bp) and assembled *de novo* (for Haloarcula marismortui, 121,886 sequencing reads with a mean length of 10,485 bp that gave a 1.278-Gb total sequence; for Haloferax mediterranei, 47,722 reads with a mean read length of 13,138 nucleotides yielding a 626-Mb total sequence) using HGAP_Assembly.1 version 2.1.1 (P4-C2 sequence) and HGAP_Assembly.3 version 2.3.0 (P6-C4 sequence), respectively, with default quality and read length parameters and polished using Quiver (5). Error correction and closure were performed using RS_BridgeMapper.1 using the default parameters, and methylation patterns were determined using RS_Modification_and_Motif_Analysis.1 within SMRT Analysis using default settings (minimum modification QV, 30).

Genome annotation was performed using the NCBI Prokaryotic Genome Annotation Pipeline (PGAP) build 3190 (6) and analyzed on the Haloarchaeal Genomes Database (HaloWeb version r1555192846) (7) using an installation of Lagan version 2.0 for comparison to the originally reported sequences (cf. https://halo.umbc.edu/cgi-bin/haloweb/hma.pl?operation=pairwise_comparator and https://www.ncbi.nlm.nih.gov/genome/1084?genome_assembly_id=300522; cf. https://halo.umbc.edu/cgi-bin/haloweb/hme.pl?operation=pairwise_comparator, https://www.ncbi.nlm.nih.gov/genome/11181?genome_assembly_id=227368, and https://www.ncbi.nlm.nih.gov/genome/11181?genome_assembly_id=173551) (8). The genome sequences were very similar, with primarily single-nucleotide indels of G (or C) in short runs of G’s (or C’s) and a few insertion element indels. The *H. marismortui* genome was 4,184,929 bp long with a 62.4% GC content and was divided into a 4.2-Mbp chromosome and 6 plasmids of 33 to 411 kbp. The H. mediterranei genome was 3,907,473 bp with a 61.1% GC content and was divided into a 3.9-Mbp chromosome and 3 plasmids of 132 to 505 kbp. Like those of other *Haloarchaea* species, the *H. marismortui* and H. mediterranei genomes encode acidic proteomes with average protein pI values of 4.7 and 4.9, respectively (9). Both genomes encode all 799 conserved haloarchaeal orthologous groups (HOGs), including 77 signature-unique conserved HOGs characteristic of the family (10).

Both the *H. marismortui* and H. mediterranei genomes contain the methylated sequence ^m4^CTAG. The corresponding type II-β methyl transferases (MTase) are coded by *zim* (VNG1543) in the model species *Halobacterium* sp. strain NRC-1 and over 100 other *Haloarchaea* species and are recognized as HOG1170 (10–13). Despite the wide distribution of CTAG methylation in the family, the function of this methylation system is still not known (14).

The *H. marismortui* and H. mediterranei genomes each also contain a single type II-α MTase enzyme forming m4C (Table 1). For *H. marismortui*, the enzyme is coded on a 155-kbp plasmid (pNG600/pHMA155), while for H. mediterranei, the enzyme is coded on the large chromosome.

**TABLE 1:** Methylated motifs in Haloarcula marismortui and Haloferax mediterranei detectable by SMRT sequencing

Species and motif^*a*^	Type	% motif detected^*b*^	Putative responsible MTase^*c*^
*H. marismortui*			
**C**TAG	4mC, II-β	89	M.HmaHMAI (ORF Hma_11876)
TCGA**C**GG	4mC, II-α	85	M.HmaHMAII (ORF Hma_6187)
H. mediterranei			
**C**TAG	4mC, II-β	97	M.Hme33500I (ORF HFX_760)
HG**C**WGCK	4mC, II-α	83	M.Hme33500II (ORF HFX_3001)

a Locations of methylated bases are bold for the top strand and underlined for the bottom strand.

b % motif detected is a specific parameter of the SMRT motif and modification software and is dependent on a number of parameters, especially the genome coverage and nature of the modified base (m6A or m4C). For m4C detection, it almost never reaches 100% even when modification is 100%.

c Putative responsible MTase is the REBASE name for the methyltransferase responsible for the modification in the first column, with the open reading frame (ORF) number identified in parentheses. It was identified by sequence comparison with known methyltransferases of that specificity in the gold standard set in REBASE and the absence of other candidates in the genome.

### Data availability.

The *H. marismortui* genome sequence has been deposited in GenBank with the accession numbers CP039132, CP039133, CP039134, CP039135, CP039136, CP039137, and CP039138. The H. mediterranei genome sequence has been deposited in GenBank with the accession numbers CP039139, CP039140, CP039141, and CP039142. Both genomes are also available on HaloWeb (https://halo.umbc.edu/) and have been analyzed in the Restriction Enzymes Database (REBASE) (http://rebase.neb.com/rebase/rebase.html) (15). Raw data are available in the NCBI Sequence Read Archive with the accession numbers SRR8914802, SRR8914803, SRR8985819, and SRR8985820.
